# Effects of audiovisual temporal synchronization on visual experience of the non-dominant eye

**DOI:** 10.1007/s10339-025-01296-3

**Published:** 2025-08-22

**Authors:** Hikari Takebayashi, Yuji Wada

**Affiliations:** 1 Ritsumeikan-Global Innovation Research Organization, Ritsumeikan University, Noji-Higashi 1-1-1, Kusatsu-shi, Shiga 525-8577 Japan; 2 College of Gastronomy Management, Ritsumeikan University, Noji-Higashi 1-1-1, Kusatsu-shi, Shiga 525-8577 Japan

**Keywords:** Audiovisual interaction, Auditory cue, Dichoptic stimulation, Eye dominance

## Abstract

Audiovisual integration occurs automatically and affects visual processing. This study aims to investigate whether temporally synchronized auditory signals enhance monocular signals during binocular observation. In Experiment 1, 16 participants performed a visual target localization task. A mirror stereoscope was used to present a rapid serial visual presentation (RSVP) stream of distractors to both eyes, with a visual target inserted in either both eyes, the dominant eye, or the non-dominant eye. Continuous low tones synchronized with distractors were paired with the target as either the same low tone (non-salience) or a high tone (salience). Detection facilitation rates by tone type were analyzed through multiple comparisons. Results showed a significant detection enhancement only when the target appeared in the non-dominant eye. In Experiment 2, involving 16 participants, a similar RSVP was presented, but with an orientation discrimination task for parafoveally presented texture stimuli comprising 17 vertical Gabor patches. The angle and proportion of tilted patches were manipulated simultaneously, and logistic regression was used to estimate orientation discrimination thresholds. Contrary to predictions, salient tones did not reduce the thresholds. These findings suggest that temporally synchronized auditory signals can selectively enhance the monocular processing of weaker visual signals (i.e., non-dominant eye signals) before binocular fusion, particularly for spatial localization. However, these effects did not extend to the identification of visual content (i.e., orientation) or stable visual signals (i.e., dominant or binocular). The results highlight the role of audiovisual integration in supporting unstable monocular signals and suggest potential applications in low vision training.

## Introduction

Integrating information across multiple sensory modalities can enrich perceptual experiences. Temporal and spatial synchrony of audiovisual information helps navigate information sources, thereby increasing the reliability of source estimation and facilitating a coherent understanding of the visual environment. Previous research revealed that the advantages of audiovisual interaction are maximized when both temporal synchrony and spatial consistency are present (Meredith and Stein 1986; Meredith et al. 1987). For instance, the timing and location of auditory stimuli can enhance visual stimulus detectability (Spence and Driver 1997), shorten response times to visual stimuli at specific locations (Mclntire et al. 2010; Perrott et al. 1991; Simon and Craft 1970), and determine the visual direction of motion (Alink et al. 2012; reviewed in Chaplin et al. 2018; Hidaka et al. 2009; Hidaka et al. 2011; Maeda et al. 2004; McCourt and Leone 2016). Moreover, in patients with hemianopia due to stroke or cortical injury, audiovisual training lasting more than 10 weeks (two hours per session) can restore flash detection in the blind visual field (Rowland et al. 2023), possibly through plasticity from subcortical (e.g., superior colliculus) to cortical regions (e.g., auditory and visual cortices) (Meredith and Stein 1983; Wallace et al. 2004).

Nevertheless, visual experiences can be enhanced through temporal synchrony even without requiring spatial correspondence between audiovisual stimuli. For instance, in a visual search task where the colors of a target and some distractors switch at regular intervals, the detection time for a visual target can be shortened by synchronizing the color changes with an auditory stimulus (Van der Burg et al. 2008). This phenomenon, known as the “pip and pop effect,” occurs when the timing of color changes in some stimuli coincides with an auditory stimulus (a pip sound) presented binaurally, leading to a pop-out effect. Preceding auditory stimuli can function as warning signals. While warning sounds may reduce reaction time, they can sometimes increase detection errors (Han and Proctor 2022; Simon et al. 1975). However, according to the pip and pop effect, the smaller the temporal gap between audiovisual stimuli, the shorter the detection time for visual targets. Therefore, it is suggested that the combination of audiovisual information enhances the subjective salience of visual targets. Because the auditory stimuli in this case do not function as warning signals, audiovisual integration is probably automatic and feedforward (Salselas et al. 2024). Relatedly, in a rapid serial visual presentation (RSVP), if a salient auditory stimulus is temporally synchronized with a visual target inserted in the stream, the visual target can appear to “freeze,” thereby enhancing the accuracy of its spatial localization (i.e., the freezing phenomenon; Vroomen and De Gelder 2000). Previous studies suggest that, rather than merely directing attention to a specific visual stimulus, the auditory signal automatically enhances visual salience.

However, the pip-and-pop effect, freezing phenomenon, and audiovisual training for patients with hemianopia all assume that auditory stimuli enhance the salience of binocular visual representations. It remains unclear whether auditory signals can enhance monocular visual signals before binocular fusion. To address this, we focused on the difference between the dominant and non-dominant eye in individuals with normal vision (Porac and Coren 1976; Rice et al. 2008), where the non-dominant eye’s signals are often underweighted or ignored in perception. Thus, we aimed to investigate whether normally suppressed visual experiences from the non-dominant eye could be activated solely through temporal synchrony with auditory signals. This may have implications for stabilizing binocular vision in cases of monocular impairment prior to cortical processing.

We conducted two RSVP tasks, presenting visual targets to both eyes, the dominant eye only, or the non-dominant eye only, while presenting distractors to the other eye, during the audiovisual stream. Stimuli were paired with low-pitched tones, while targets were synchronized either with the same low tone or a high-pitched tone. We hypothesized that salient high-pitched tones would independently enhance monocular visual signals, predicting detection rates similar to binocular presentation. Alternatively, if auditory signals contribute only to binocular processing, detection rates would be lower for monocular presentations, particularly for the non-dominant eye, due to its inherently unstable representation. This study examined whether auditory signals influenced “where” information in a localization task (Experiment 1) and “what” information in an orientation discrimination task (Experiment 2).

## Experiment 1

A four-alternative forced choice (4AFC) procedure was used for a localization task involving a visual target inserted during an RSVP. We examined whether localization was enhanced by audiovisual temporal synchronization. We compared the performance across the three presentation conditions: both eyes, the dominant eye, and the non-dominant eye.

### Materials and methods

#### Participants

Twenty observers from Ritsumeikan University participated in this study (seven men and 13 women; age range: 19–35 years). All the participants had normal or corrected-to-normal vision and no relevant medical history. They first provided written informed consent. After the experiment, the participants received a gift certificate worth JPY 1,000 for their 1 h of participation. The study was approved by the Institutional Review Board of the Ethics for Research Involving Human Subjects at Ritsumeikan University.

#### Apparatus and stimuli

The experiments were conducted individually in a dark room. The participants sat at 47 cm from a 31.1-inch liquid crystal monitor (ColorEdge CG318, EIZO Corporation, JAPAN) with a 60-Hz refresh rate, 1920 × 1080 resolution, and 40 cd/m^2^ luminance. An ophthalmic chin and forehead rest was used for head positioning. The participants viewed the left and right screens with their left and right eyes, respectively, through a mirror stereoscope, as the screen was divided into two sections (NAMOTO, Co. Ltd., JAPAN). The preparation and presentation of the visual stimuli were controlled using GNU Octave 7.3.0 (GNU General Public License) with the Psychtoolbox extension (Brainard 1997).

**Fig. 1:**
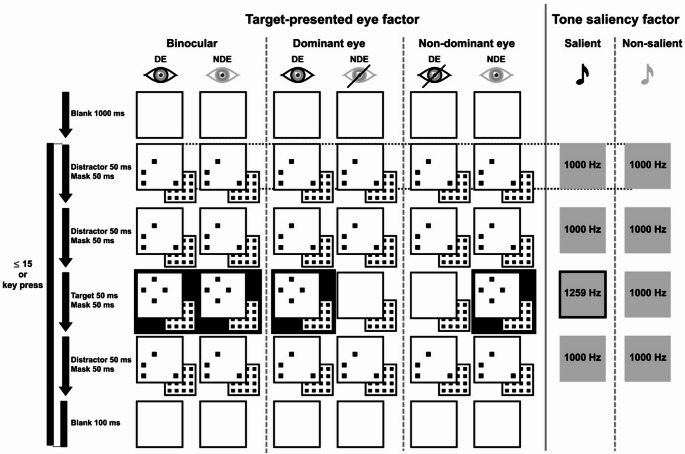
Experimental design and flow diagram of a single trial in Experiment 1

All the visual stimuli were presented within a white square subtending 4.5° in the visual angle at the center of a black screen. The white square was always presented as a frame to stabilize binocular fusion. Within this square, 4 × 4 matrix placeholders were virtually created. One distractor comprised four black dots randomly placed from the 16 placeholders (Fig. 1). One target comprised four dots forming a diamond shape and was presented at one of the four corners inside the square: top-left, top-right, bottom-left, or bottom-right. In addition, mask stimuli comprising dots drawn from all 16 placeholders were created. Each dot measured 4 × 4 pixels. Auditory stimuli consisted of pure tones at frequencies of 1000 Hz and 1259 Hz (four semitones higher) with a 44,100 Hz sampling rate. The intensity of the sound was approximately 60 dB SPL to clearly distinguish each tone. The sound was always presented to both ears via headphones (AKG Q701, Harman International Industries, Inc., Stamford, USA).

Four test displays, consisting of three distractors and one target followed by masks, were presented in a visual stream. Each distractor comprised four dots positioned randomly within 16 placeholders, whereas the target comprised four dots forming a diamond shape. This stream was looped up to 15 times in a single trial. Visual stimulation was performed dichoptically using a mirror stereoscope. The visual target in the third display was presented to both eyes, the dominant eye, or the non-dominant eye. The distractors were identical throughout a single trial, but they were randomized between trials. Auditory stimulation was synchronized with each test display for 50 ms, except for masks. The tone sequence was conducted under two conditions: salient and non-salient. DE: dominant eye, NDE: non-dominant eye.

#### Procedure of dominant eye test

**Fig. 2:**
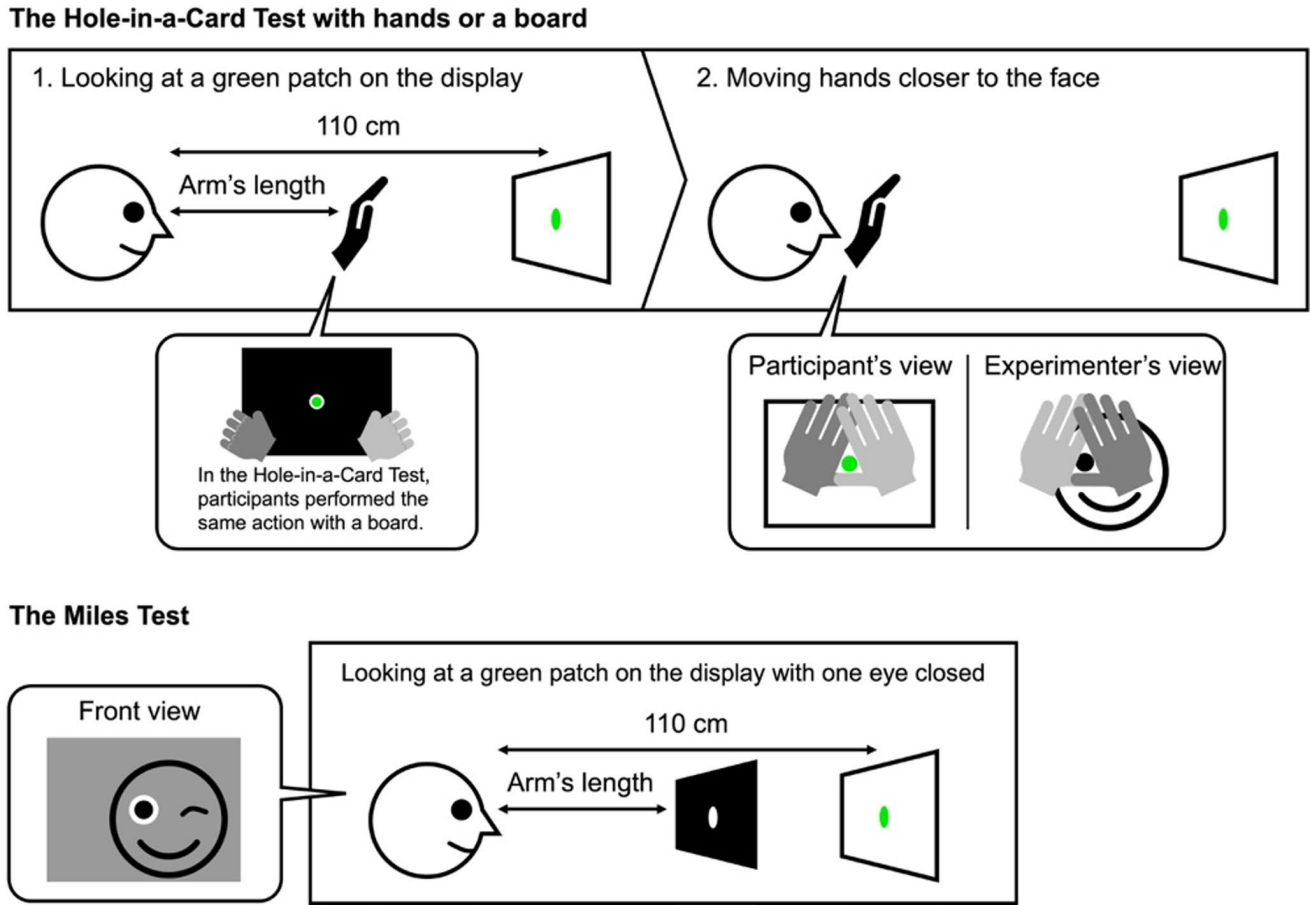
Sighting-dominant eye test procedure

Before entering the dark room, each participant performed three sighting-dominant eye tests in a well-lit room. The first test was the Hole-in-a-Card Test, where participants used a 21.6 × 30 cm board with a hole that was 3 cm in diameter in the center (Fig. 2). Holding the board at arm’s length, the participants peered at a green patch displayed on a monitor 110 cm away through the hole using both eyes. The patch, subtending 2.2° in the visual angle, fit exactly into the hole. The participants were instructed to slowly bring the board towards their face while maintaining fixation on the patch. If the position of the hole shifted horizontally towards one eye when the board was brought in front of their faces, the experimenter identified that eye as the dominant eye. Most participants were not conscious that the final board position had shifted to one eye because they believed that they had observed the patch using both eyes. Next, the participants formed a small triangle by overlapping their hands and repeated the same action as in the first test (Fig. 2). The eye aligned with the direction of the shifted hand was identified as the dominant eye. The last test was the Miles Test, which used the same board as in the first test. The participants held the board at arm’s length, peered at the patch through the hole, and alternately closed one eye. The dominant eye was identified based on the open eye that successfully captured the patch. Using these three tests, we determined the final dominant eye based on the majority score.

The Hole-in-a-Card Test consists of two steps. First, participants stretch their arms and create a small triangle using both hands. While watching a green patch through the triangle, the participants slowly bring both hands to their faces. The eye that keeps fixating and induces a subtle horizontal shift of the triangle (i.e., both hands) is defined as the sighting-dominant eye. Again, participants use a board truncated at the center and perform the same procedure. In the Miles Test, participants use the same board and observe the patch through the hole with one eye closed alternately. The eye capturing the patch in the hole is the dominant one.

#### Procedure of location detection task

Following the sighting-dominant eye tests, participants received instructions for the main task through an oral explanation and slide presentation. Additionally, we presented four trials of an audiovisual stream at a much slower tempo than the actual task to ensure their comprehension. After confirmation, the participants entered a dark room to perform 10 practice trials at the actual tempo. Before starting the practice, the participants reconfirmed a brief text of instructions on the screen, which also helped with binocular fusion. If the participants experienced discomfort with the binocular fusion of the text, the experimenter instructed them to rotate the mirrors on either side until the images merged. A trial started with a blank screen featuring only a white square on a black background for 1000 ms. Subsequently, four displays comprising four dots were presented with immediately accompanying masks. A visual target (diamond formation) was inserted as the third display (see Fig. 1). Each of the four-dot displays and masks was presented for three frames (50 ms), resulting in a total duration of 400 ms for these eight displays. Because a blank screen was presented for six frames (100 ms) after the series of eight displays, the total duration was 500 ms. It looped for a maximum of 15 times, with a duration of 7500 ms. However, the participants were instructed to press the corresponding key immediately upon perceiving the target’s position during the loop; thus, the actual duration was often shorter than 15 loops. If no response was elicited within 15 loops, the program automatically transitioned to the next trial. Participants’ responses were recorded using two numeric keypads (BSTKH08, Buffalo Inc., JAPAN, and ST-U2NK, SATECHI, CA, USA) placed on either side of the mirror stereoscope. The “7” and “4” keys on the left keypad corresponded to the top-left and bottom-left responses, whereas the “-” and “+” keys on the right keypad corresponded to the top-right and bottom-right responses, respectively. To prevent erroneous input, all other keys were disabled during the task. The four-dot displays were synchronized with auditory stimuli consisting of four low (L) tones at 1000 Hz (LL“L”L) or four tones including a high (H) tone at 1259 Hz in the third display (LL“H”L).

Furthermore, another factor involved presenting visual targets to both eyes, the dominant eye alone, or the non-dominant eye only. For the latter two conditions, a blank screen was presented to the other eye on the third display, whereas the flow of the other displays remained the same for both eyes. This manipulation was based on the pilot results from two volunteers in our laboratory and the author (HT). The pilot experiment involved a parallel presentation of the target to one eye and a mask (16 dots) to the opposite eye on the third display. However, this composition was assumed to be difficult because the results showed generally low detection rates approaching chance levels. Conversely, the blank screen inserted in the third display in the opposite eye did not cause any discomfort to the observers during visual stimulation. The procedure in this task was similar to that used in a previous study (Vroomen and De Gelder 2000), but with a faster presentation time per display and no warm-up period. This was based on the three pilots’ data. The number of responses over 15 loops was 6.7% of all trials in the valid data, suggesting that participants became accustomed to the sequence within 4–8 loops without any special warm-up period.

Three within-subject factors were examined: two levels of the tone saliency (salient and non-salient), three levels of the target-presented eye (binocular, dominant eye, and non-dominant eye), and four levels of the target position (top-left, top-right, bottom-left, and bottom-right). This combination generated 24 subconditions with 16 repetitions. All conditions were randomized, with a short break inserted every 48 trials, for a total of 384 trials.

### Results and discussion

Because data from four participants with overall low detection rates were excluded from the analysis, 16 datasets were analyzed. The criterion was defined as whether the average detection rate fell below the chance level (25%) across the three target-presented eye conditions in the non-salient tone condition, as it served as a reference for comparison between the tone saliency conditions.

**Fig. 3:**
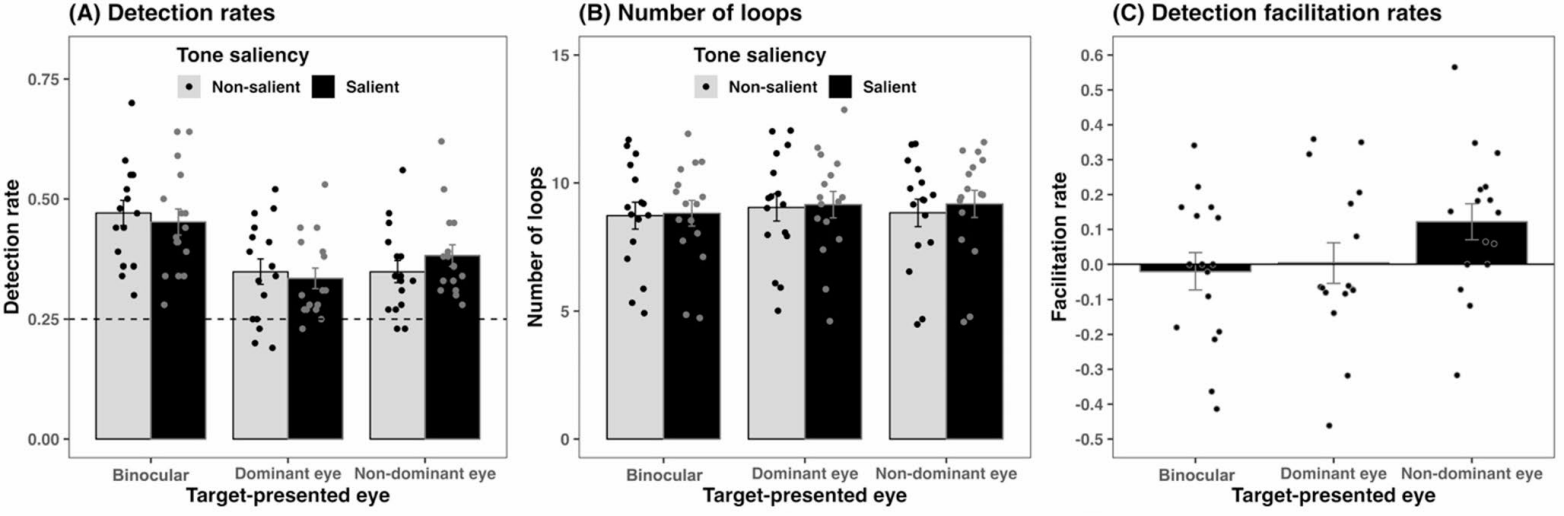
Results of Experiment 1. (**A**) The average detection rate for the target across 16 participants. The horizontal dotted line represents the chance level. Each point represents an individual participant’s data. (**B**) The average number of loops until the key press is used as an index of detection time. (**C**) Average detection facilitation rates were calculated using the formula: $$\:({detection\:rate}_{salient}-$$$$\:{detection\:rate}_{non-salient})/$$$${detection\:rate}_{non-salient}$$. Error bars represent the standard error of the mean.

Figure 3 presents plots of the average detection rates, loop numbers, and detection promotion rates. All descriptive statistics are listed in Table 1. First, a repeated-measures analysis of variance (ANOVA) was conducted for detection rates, considering factors of tone saliency and the target-presented eye, on R (version 4.3.1). The alpha level was set at 0.05. Because the main effect of the target-presented eye factor was significant, *F*(2, 30) = 34.324, *p* <.001, $\:{\eta\:}_{\mathrm{p}}^{2}$ = 0.696, Bayes Factor BF_10_ = 1.935e+8, Bonferroni-corrected post-hoc comparisons revealed that the binocular condition had higher detection rates than the dominant eye, *t*(15) = 7.819, *p* <.001, Cohen’s d = 1.955, BF_10_ = 557644.011, and the non-dominant eye conditions, *t*(15) = 6.284, *p* <.001, Cohen’s d = 1.571, BF_10_ = 104686.232. There was no significant difference between the monocular conditions, *t*(15) = 1.535, *p* =.406, Cohen’s d = 0.384, BF_10_ = 0.612. Furthermore, neither the main effect of the tone saliency factor, *F*(1, 15) = 1.924e−4, *p* =.989, $\:{\eta\:}_{\mathrm{p}}^{2}$ = 1.283e−5, BF_10_ = 0.215, nor the interaction, *F*(2, 30) = 2.030, *p* =.149, $\:{\eta\:}_{\mathrm{p}}^{2}$ = 0.119, BF_10_ = 3.972e+7, was significant (Fig. 3A).

**Table 1:** Quantitative data of experiment 1

Target-presented eye	*N*	Tone	Detection rate	(SD)	Loop numbers	(SD)	Detection facilitation rates	(SD)
Binocular	16	Salient	0.45	(0.11)	8.81	(2.01)	−0.02	(0.21)
		Non-salient	0.47	(0.11)	8.72	(2.11)		
Dominant eye	16	Salient	0.34	(0.09)	9.15	(2.07)	0.00	(0.23)
		Non-salient	0.35	(0.11)	9.04	(2.14)		
Non-dominant eye	16	Salient	0.38	(0.09)	9.18	(2.12)	0.12	(0.21)
		Non-salient	0.35	(0.09)	8.83	(2.15)		

Detection rate, loop numbers, and detection facilitation rates represent mean value across 16 participants. *N*: the number of participants, *SD*: standard deviation.

However, anisotropy across the presentation conditions was observed in the detection rates as influenced by the tone salience. Specifically, the detection rate increased only under the non-dominant eye condition when the tone was salient. Therefore, we focused on the detection facilitation rate: $$(({detection\:rate}_{salient}-$$$${detection\:rate}_{non-salient})/$$$${detection\:rate}_{non-salient})$$ in relation to tone salience. Multiple comparisons with an alpha of 0.017 revealed that the detection facilitation rate for the non-dominant eye condition was significantly higher than that for the binocular condition, *t*(15) = 3.040, *p* =.008, Cohen’s d = 0.76, BF_10_ = 6.394. There were no significant differences between the non-dominant and dominant eye conditions, *t*(15) = 1.547, *p* =.143, Cohen’s d = 0.387, BF_10_ = 0.688, nor between the binocular and dominant eye conditions, *t*(15) = 0.356, *p* =.727, Cohen’s d = 0.089, BF_10_ = 0.271 (Fig. 3C).

Next, a repeated-measures analysis of variance (ANOVA) with an alpha of 0.05 revealed a significant main effect of the target-presented eye on the number of loops, *F*(2, 30) = 5.179, *p* =.012, $\:{\eta\:}_{\mathrm{p}}^{2}$ = 0.257, BF_10_ = 3.126. The number of loops indicated the reaction time for key presses (Fig. 3B). Bonferroni-corrected post-hoc comparisons revealed that the binocular condition had fewer loops than the dominant eye condition, *t*(15) = −3.122, *p* =.012, Cohen’s d = 0.780, BF_10_ = 25.765, but did not significantly differ from the non-dominant eye condition, *t*(15) = −2.240, *p* =.098, Cohen’s d = 0.560, BF_10_ = 3.014. Furthermore, there was no significant difference between the monocular conditions, *t*(15) = 0.882, *p* = 1.000, Cohen’s d = 0.22, BF_10_ = 0.265. Additionally, neither the main effect of the tone saliency factor, *F*(1, 15) = 2.033, *p* =.174, $\:{\eta\:}_{\mathrm{p}}^{2}$ = 0.119, BF_10_ = 1.063, nor the interaction, *F*(2, 30) = 1.246, *p* =.302, $\:{\eta\:}_{\mathrm{p}}^{2}$ = 0.077, BF_10_ = 3.481, was significant. In summary, participants exhibited the best performance in terms of both detection rates and loop numbers under the binocular condition, indicating that there was no speed-accuracy trade-off.

The lack of contribution from the salient tone to the detection facilitation rate in the binocular condition contradicts the findings from previous research on the freezing phenomenon. This inconsistency might be due to ceiling effects arising from the absence of trade-off or extraneous factors introduced using the mirror stereoscope. Notably, the detection facilitation rate increased only in the non-dominant eye condition from the non-salient to salient tone conditions, suggesting that the temporal synchronization of audiovisual stimuli enhances the saliency of unstable monocular signals in the localizing task.

## Experiment 2

To further investigate the conditions under which a salient auditory signal enhances visual processing in the non-dominant eye, we conducted Experiment 2, focusing on an orientation discrimination task involving a visual target in the parafovea.

### Materials and methods

#### Participants

Twenty-one observers (seven men and 14 women; age range: 19–35 years) participated in this experiment. Thirteen of them had also participated in Experiment 1. All participants had normal or corrected-to-normal vision. The informed consent, compensation, and ethical review procedures were consistent with those used in Experiment 1. Because this experiment lasted approximately 2 h, participants received a gift certificate worth JPY 2,000.

#### Stimuli

**Fig. 4:**
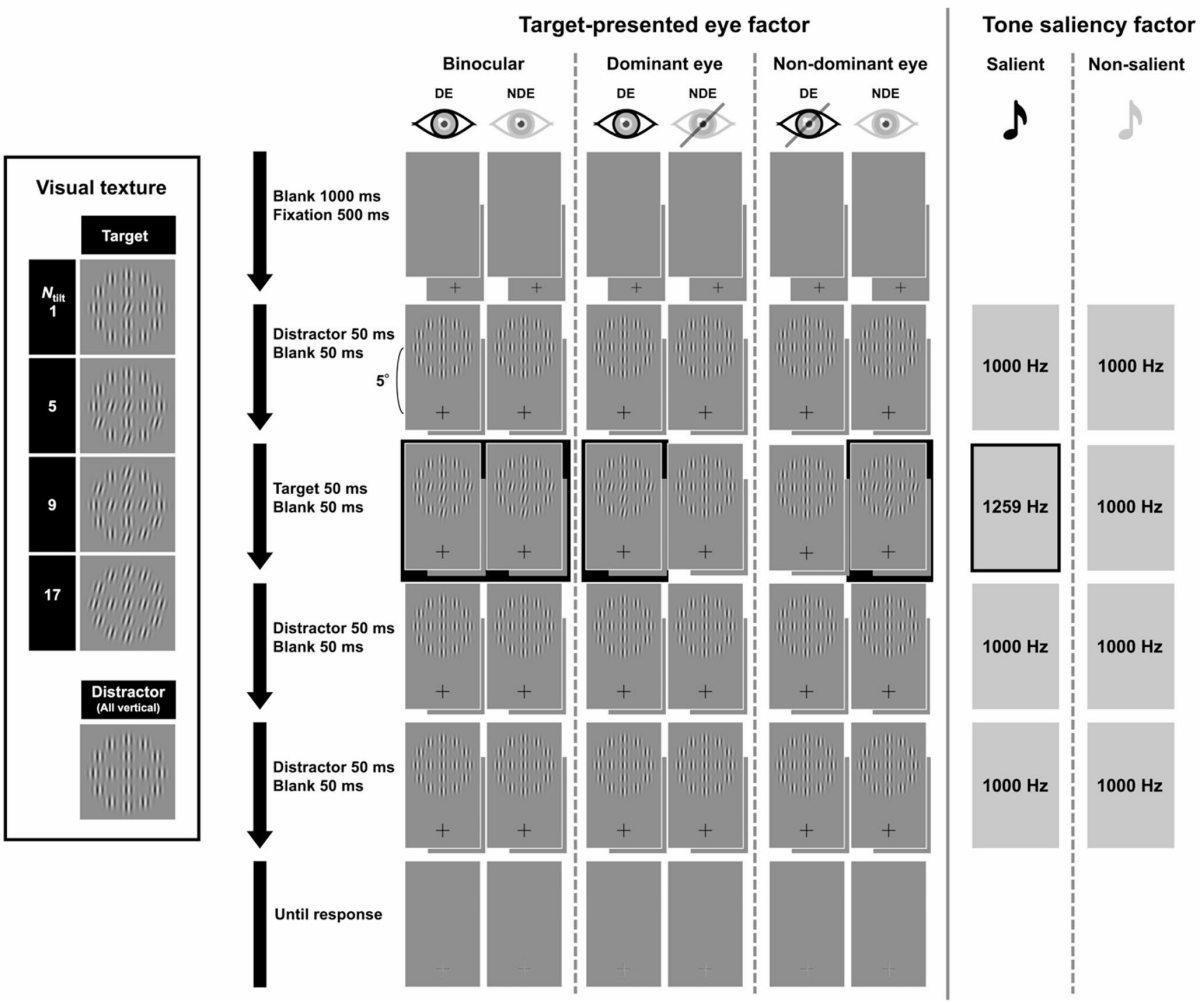
Examples of the visual textures, experimental design, and flow diagram of a single trial. (Left) Visual stimuli of textures comprising 17 Gabor patches. The visual target contains tilted patches, whereas the visual distractors comprise 17 vertical patches. The tilt angles of the target patches are ± 2°, ± 6°, ± 10°, or ± 14° from vertical. Positive values indicate clockwise tilt, whereas negative values indicate counterclockwise tilt. (Right) The sequence of audiovisual stimulation comprised a single rapid stream without loops. The visual target always appeared as the second display to promote stable fixation. Across trials, visual textures were randomly presented either above or below the center of the screen. This example shows the texture center presented 5° above the fixation point. The auditory stimuli were identical to those used in Experiment 1. *DE:* dominant eye, *NDE:* non-dominant eye.

The equipment, including the dark room, monitor, mirror stereoscope, headphones, and platform for creating and controlling experimental stimuli, was identical to those used in Experiment 1. In Experiment 2, a texture stimulus comprising 17 Gabor patches was presented on a gray background with a luminance of 2.05 cd/m^2^ (Fig. 4). Each patch had a spatial frequency of three cycles per degree (cpd), and the distance from the center of the central patch to the outermost patch was 2° of visual angle. Similar to Experiment 1, a stream of four displays consisting of three visual distractors and one target was presented in rapid succession. However, the texture appeared 5° above or below the fixation cross. The visual distractors consisted of textures with all 17 patches oriented vertically, whereas the visual targets included textures in which 1, 5, 9, or all 17 patches were tilted at the same angle. When only one tilted patch was included in the target, it was always located at the center of the texture, resulting in crowding. This refers to a perceptual phenomenon in which the recognition of a central stimulus is impaired by surrounding stimuli when the set of stimuli is in the parafovea (Freeman and Simoncelli 2011; Freeman et al. 2013; Parkes et al. 2001). When five or nine patches were tilted, their locations were randomized on each trial, and the patches were tilted at ± 2, 6, 10, or 14° from vertical. The auditory stimuli were the same as those in Experiment 1.

#### Procedure

Similar to Experiment 1, each participant completed dominant eye tests, received task instructions, and performed 10 practice trials before beginning the main trials. Each trial started with a blank screen for 1000 ms, followed by a fixation cross displayed for 500 ms. Next, three visual distractors and one target were each presented consecutively for three frames (50 ms). Throughout the stream, the fixation cross remained at the center of the screen, while the four displays were presented 5° above or below the fixation cross. Unlike in Experiment 1, the visual target was always presented as the second display in the stream. Instead of using a mask, each stimulus was followed by a blank screen for three frames, and no looping occurred within a trial. The target was placed second to ensure that participants maintained fixation, which was essential for investigating the crowding effect. Additionally, blank screens were used instead of masks because pilot experiments conducted by the experimenter (HT) indicated an overall accuracy below 55%, rendering the initial mask design unsuitable. Therefore, each trial consisted of a 400 ms stream, comprising four textures displays and four accompanying blank screens. As in Experiment 1, the auditory stimuli were synchronized with the visual distractors as low tones and with the target stimulus as either a low (L“L”LL) or a high tone (L“H”LL). After the stream, the fixation cross turned green, prompting participants to respond using the right (“RB”) and left (“LB”) buttons on a controller (WOLVERINE V2 CHROMA, Razer, CA, USA) to indicate whether the texture tilted clockwise or counterclockwise relative to vertical.

The main task comprised 1920 trials, consisting of 192 subconditions with 10 repetitions each. These subconditions were created by combining two levels of the tone saliency (salient and non-salient), three levels of the target-presented eye (binocular, dominant eye, and non-dominant eye), four levels of the number of tilted elements (1, 5, 9, and 17), and eight levels of the tilt angle (± 2, 6, 10, and 14°). Unlike a blocked design, all subconditions were randomly presented during the task, with a short break provided every 192 trials.

### Results and discussion

**Fig. 5:**
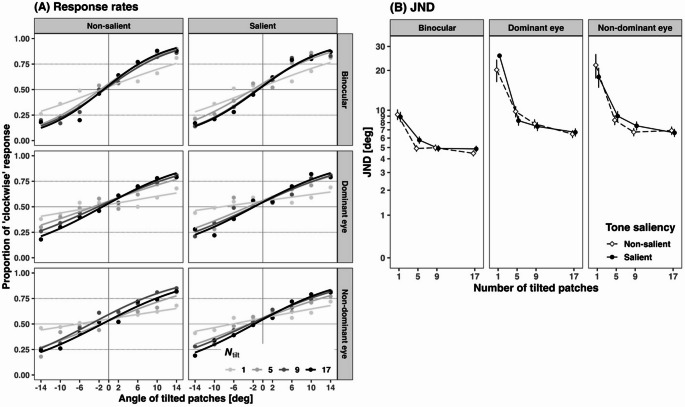
Results of Experiment 2. (**A**) Psychometric curves of “clockwise” response rates depending on the angle of tilted patches, with separated lines per the number of tilted patches (*N*_tilt_). Columns represent the tone saliency factor, while rows represent the target-presented eye factor. (**B**) The JND is calculated using the 25th, 50th, and 75th percentiles of the curves. Error bars represent the standard error.

To exclude outliers, we applied a criterion based on whether the participant’s average accuracy fell below 55% in the non-salient condition, which served as a reference for assessing the effect of auditory synchronization. Accuracy levels approaching the chance level (50%) indicated poor overall discrimination sensitivity. Based on this criterion, data from five participants were excluded from the analysis. The remaining 16 participants’ data were pooled for the logistic regression analysis. Figure 5A plots the response rates across tilt angles and exhibits a steeper curve for the binocular conditions compared to the monocular conditions, indicating higher discrimination sensitivity. In each of the 12 subconditions, defined by combinations of the target-presented eye and the number of tilted patches, we calculated discrimination thresholds (just noticeable difference: JND) using the 25th, 50th, and 75th percentile. The JND values represent the minimum tilt angles required to accurately discriminate the texture orientation in the parafovea. Figure 5B shows the JND plots for each tilted patch. The thresholds in all conditions are listed in Table 2. A logistic regression analysis was conducted using R (version 4.3.1), incorporating the four factors: tone saliency, target-presented eye, number of tilted patches, and tilt angle. The alpha was 0.05. Table 3 presents the statistical details of all conditions.

Regarding the target-presented eye factor, the binocular condition had a significantly steeper curve than the dominant eye, *z* = −12.144, *p* <.001, odds ratio (OR) = 0.695, 95% confidence interval (CI) (0.655, 0.737), and non-dominant eye conditions, *z* = −10.654, *p* <.001, OR = 0.726, 95% CI (0.684, 0.770). However, there was no significant difference between the monocular conditions, *z* = 1.503, *p* =.133, OR = 1.045, 95% CI (0.987, 1.106).

**Table 2:** Quantitative data of experiment 2

Target-presented eye	*N*	Number of tilted patches	Threshold (deg)			
			Salient tone	(*SD*)	Non-salient tone	(*SD*)
Binocular	16	1	8.89	(−28.32)	9.26	(30.47)
		5	5.77	(−13.55)	4.91	(10.54)
		9	4.92	(−10.52)	4.98	(10.72)
		17	4.87	(−10.36)	4.46	(9.14)
Dominant eye	16	1	25.67	(−217.19)	20.19	(134.78)
		5	8.29	(−25.07)	9.77	(33.78)
		9	7.45	(−20.71)	7.83	(22.62)
		17	6.68	(−17.21)	6.47	(16.27)
Non-dominant eye	16	1	17.88	(−106.83)	21.89	(158.39)
		5	9.00	(−29.07)	8.37	(25.40)
		9	7.54	(−21.20)	6.71	(17.51)
		17	6.62	(−16.94)	6.91	(18.16)

The discrimination threshold was calculated by pooling data from 16 participants. *N:* the number of participants, *SD:* standard deviation.

Figure 5A shows that an increase in the tilted patches significantly affected the steepness of the logistic curve, *z* = 10.382, *p* <.001, OR = 1.416, 95% CI (1.326, 1.512) for *N*_tilt_ = 1 vs. 5, *z* = 3.573, *p* <.001, OR = 1.131, 95% CI (1.057, 1.211) for *N*_tilt_ = 5 vs. 9, *z* = 2.747, *p* =.006, OR = 1.102, 95% CI (1.028, 1.180) for *N*_tilt_ = 9 vs. 17 (see other statistical values in Table 3). As shown in Fig. 5B, JND also decreased monotonically with an increasing number of tilted patches. These results align with those of previous research on crowding (Freeman and Simoncelli 2011; Freeman et al. 2013; Parkes et al. 2001). In the peripheral visual field, local signals are pooled, causing observers to lose the ability to perceive the shape of a central stimulus within a group. Conversely, the pooling of local orientation signals within a texture implies that increasing number of tilted patches strengthen the global orientation signal of the texture. Although there may be concerns that presenting the target stimulus to one eye and vertically oriented texture to the other could induce a perception of depth tilt, the monotonic decrease in JND with an increasing number of tilted patches suggests that participants correctly judged the orientation on the frontal plane. However, tone saliency did not affect the curve, *z* = −0.692, *p* =.489, OR = 0.983, 95% CI (0.938, 1.031), indicating that the insertion of high tones did not facilitate the visual discrimination of monocularly presented target stimuli in the texture orientation task. This suggests that auditory signals did not enhance the clarity of visual content.

**Table 3:** Statistical values of experiment 2

Factor	Comparison		Coefficient	z	OR	OR 95% CI		*p*	
	Reference	Objective				Lower	Upper		
Tone saliency			−0.02	−0.69	0.98	0.94	1.03	0.49	
Target-presented eye	Binocular	Dominant eye	−0.36	−12.14	0.69	0.65	0.74	< 0.001	***
	Binocular	Non-dominant eye	−0.32	−10.65	0.73	0.68	0.77	< 0.001	***
	Dominant eye	Non-dominant eye	0.04	1.50	1.04	0.99	1.11	0.13	
Number of tilted patches	1	5	0.35	10.38	1.42	1.33	1.51	< 0.001	***
	1	9	0.47	13.91	1.60	1.50	1.71	< 0.001	***
	1	17	0.57	16.60	1.77	1.65	1.89	< 0.001	***
	5	9	0.12	3.57	1.13	1.06	1.21	< 0.001	***
	5	17	0.22	6.31	1.25	1.16	1.33	< 0.001	***
	9	17	0.10	2.75	1.10	1.03	1.18	0.006	**
Angle of tilted patches			0.55	17.36	1.73	1.63	1.85	< 0.001	***

*z* and *p* values are calculated using logistic regression analysis. *OR:* odds ratio, *CI*: confidence interval. ^**^p <.01, ^***^p <.001

## General discussion

The results from the two experiments reveal that synchronized salient tones can modestly enhance the detection of target location when presented to the non-dominant eye, but they do not facilitate orientation discrimination. The effect of audiovisual temporal synchronization on visual localization tasks aligns with the “pip and pop” effect and “freezing phenomenon” (Van der Burg et al. 2008; Vroomen and De Gelder 2000). A major difference between previous studies is that our findings demonstrate that auditory signals can selectively influence monocular signals. The selective effect on the non-dominant eye, observed solely in the localization task, may reflect its greater susceptibility to correction by external stimuli (Money 1972; Walls 1951). In other words, the non-dominant eye, being relatively unstable in the visual fixation and generation of perceptual representations, might be more amenable to corrections. Conversely, because the dominant eye is specialized for the stable capture of visual stimuli, as observed in dominant eye tests, it might also be less susceptible to corrections by external factors. In other words, the visual information coming from the dominant eye is likely to be represented stably on its own.

In Experiment 2, which involved discriminating the visual orientation in the parafoveal region, temporal synchronization of audiovisual stimuli did not yield any observable benefit. While speculative, this finding warrants consideration from a neuroscientific perspective. The existence of multisensory neurons has been confirmed not only in the superior colliculus (Meredith and Stein 1986; Meredith et al. 1987; Wallace et al. 1996) but also in the cortex. Anatomical studies have shown an eccentricity-dependent gradient in the neural connections between the primary visual cortex (area 17/V1) and primary auditory cortex (AC) (Falchier et al. 2002; Mazo et al. 2024). This gradient may not effectively support the processing of “what” information in vision. Specifically, among primates, AC projections primarily target peripheral visual field representations (> 10°) in V1, while projections to foveal and parafoveal areas (0–8°) are minimal. Similarly, the density of projection neurons from the temporal parieto-occipital area to V1 increases with eccentricity, paralleling the gradient of AC-derived projections. This suggests that projections from AC to V1 are better suited for spatial localization and for inducing eye movements toward peripheral stimuli, rather than for enhancing the encoding of visual content. If auditory signals were intended to enhance visual content encoding, one would expect comparable projections to neural populations in both the foveal and peripheral visual fields. However, considering that shape perception in the parafovea is generally robust (except under specific conditions, such as crowding), the lack of an effect of salient auditory signals on “what” information in this study is not surprising.

Ocular dominance can be subdivided into sensory dominance and sighting dominance (Pointer 2012). Additionally, some tasks may involve motor dominance as a subclass (Ooi and He 2020). Sensory dominance refers to the monocular signal that perceptually dominates, such as in binocular rivalry, where one eye’s representation suppresses the other. Sighting dominance indicates the eye preferentially used for alignment when fixating on a target, which was measured in the current study’s dominant-eye tests. Motor dominance refers to the eye that primarily guides movement when tracking a moving object. Although the eyes typically move in coordination, during vergence movements (convergence or divergence), one eye may maintain fixation while the other “gives up” its fixation. In this study, it is possible that the salient sound affected sensory dominance during the experiments. However, given the organic link between these subcategories, it cannot be ruled out that the integration of auditory signals with unstable visual signals might enhance the sensory level and subsequently influence the sighting level in a retroactive manner. Our experimental paradigm did not allow us to determine whether “sighting alignment” preceded the enhancement of sensory representations in audiovisual interactions. Considering this limitation, future studies should explore the behavioral characteristics underlying the enhancement of monocular signals by measuring eye movements. Furthermore, with respect to potential applications, low vision care is one of the most important fields. For amblyopia, patching therapy is typically used to make participants use a weaker eye by covering a better eye. However, while it can improve vision in the weaker eye, it often does not contribute to stereopsis because it does not involve coordinated binocular movements (Wallace et al. 2011). Therefore, using audiovisual temporal synchrony, it may be possible to selectively enhance monocular signals while presenting simultaneous visual input to both eyes, as demonstrated in this study. This could contribute to stabilizing the binocular visual experience as a new training paradigm.

## Conclusions

We demonstrated that the temporal synchronization of salient auditory signals with monocular visual targets enhances the detection of target positions. However, this synchronization did not affect orientation discrimination in the parafovea. Thus, this audiovisual integration likely functions to increase the salience of stimuli for spatial localization (“where” information), rather than elaborating shape representations. Furthermore, given that the enhancement of position detection was particularly pronounced for the non-dominant eye, these findings suggest the presence of a mechanism that selectively reinforces unstable monocular signals prior to binocular fusion.

## Data Availability

Experimental data and images of the experimental stimuli related to this article can be found online at https://osf.io/a54zx/?view_only=50198ebde840404f881a821d52790d65.
